# Bilateral simultaneous sino-nasal inverted papilloma; A report of two cases and literature review

**DOI:** 10.1016/j.ijscr.2019.12.034

**Published:** 2020-01-09

**Authors:** Ali Al Momen, Haifa Lafi Alenzi, Mohammad Al Eid

**Affiliations:** a Consultant ENT, Rhinology and Skull Base Surgery at King Fahad Specialist Hospital, Dammam, Saudi Arabia; b Medical Intern, Northern Border University, Arar, Saudi Arabia; c ENT Resident, Saudi Commission of Health Specialties Eastern Province Program, Saudi Arabia

**Keywords:** IP, Inverted papilloma, CCP, Convoluted Cribriform Pattern, MRI, Magnetic Resonance Imaging, ESS, Endoscopic Sinus Surgery, EMM, Endoscopic Medial Maxillectomy, PND, Paroxysmal Nocturnal Dyspnea, Inverted papilloma, Nose, Nasal sinuses, Nasal obstruction, Endoscopic sinus surgery

## Abstract

**Introduction:**

Inverted Papilloma is a benign sinonasal tumor with a high recurrence rate and potential for malignant transformation, it typically presents as an obstructing unilateral nasal mass, atypical presentations include bilateral involvement which occurs in up to 5 % of cases.

**Case presentation:**

Here we present two different cases of bilateral inverted papilloma, both presented complaining of bilateral nasal obstruction, the second case also had associated nasal polyposis and history of multiple previous endoscopic sinus surgeries, both cases were managed with endoscopic medial maxillectomy and tumor removal, with no signs of recurrence on follow up.

**Conclusion:**

Bilateral involvement is an uncommon, atypical presentation of inverted papilloma, endoscopic surgery is a safe, reliable approach and it is the mainstay of treatment, regular endoscopic and clinical follow up is important for detection of recurrence.

## 1 Introduction

Inverted papilloma (IP) is a rare benign tumor of the nasal cavity and para-nasal sinuses composed of well-differentiated columnar or ciliated respiratory epithelium having variable squamous differentiation. It is aggressive tumor which typically presents as a unilateral lesion originating from the lateral wall of the nasal cavity; therefore, it is quite rare to be seen bilateral with a reported frequency range from 0 to 5 % [1] bilateral lesions can be classified as synchronous or Metachronous [2]. Sino-nasal papilloma were first described by Ward and Billroth in the 1850s, but it was Hyams [3] who classified IP, on the basis of its histological features [4]. The exact etiology of IP is unknown, although either chronic inflammation and human papilloma virus have been inconsistently associated [5,6]. The presentation can be similar to other sinonasal masses, with nasal obstruction, sinus pain, and epistaxis. Definitive diagnosis of Inverted Papilloma is made on histopathological examination. However, Magnetic Resonance Imaging (MRI) may show a characteristic feature described as a Convoluted Cribriform Pattern (CCP). It has a high recurrence rate and a squamous cell carcinoma association. Recent studies have demonstrated the efficacy of the endoscopic approach in management of this neoplasm [7,8]. Here we report two cases with bilateral Inverted papilloma presenting as bilateral nasal obstruction. Both were managed with bilateral endoscopic navigation-assisted medial maxillectomy. Both patients have no sign of recurrence on long term follow up.

## 2 Case presentations

### 2.1 Case 1

**Fig. 1:**
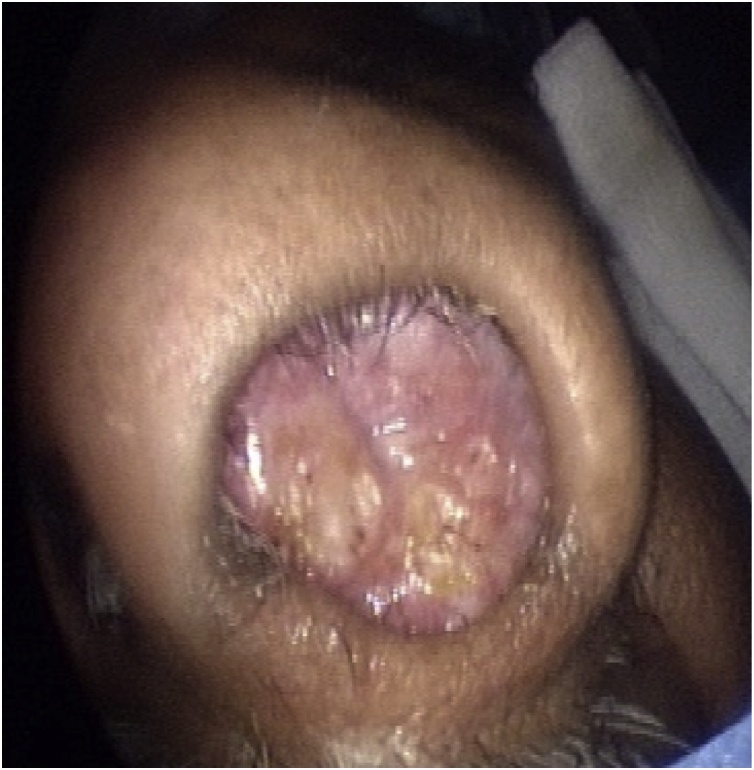
Showed external nasal deformity, bulging left lateral nasal wall, fleshy mass seen through left nostril.

**Fig. 2:**
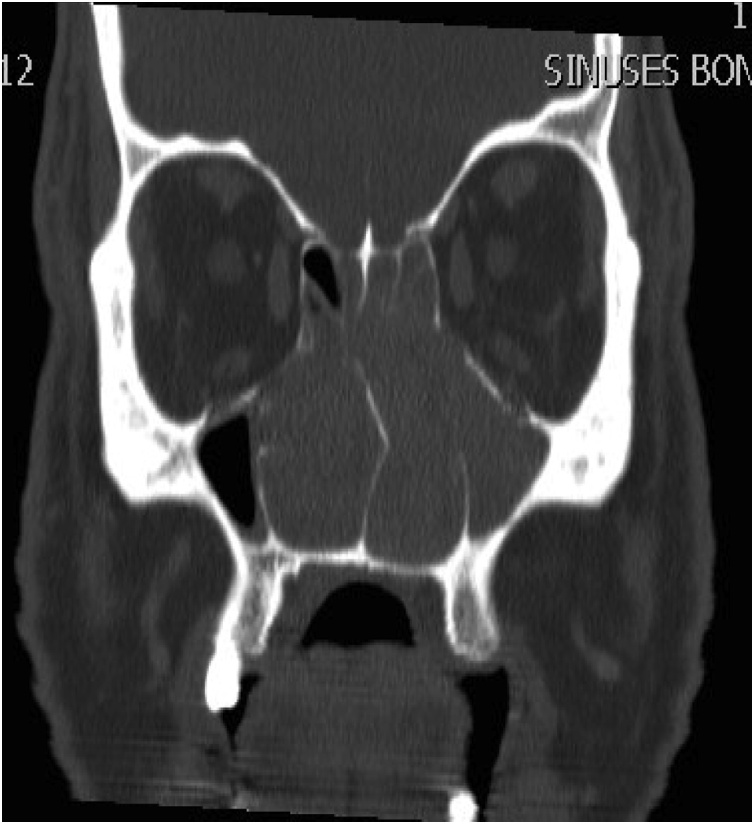
CT scan of the para-nasal sinuses showed bilateral homogenous opacity filling both nasal cavities and the left maxillary and frontal sinuses.

**Fig. 3:**
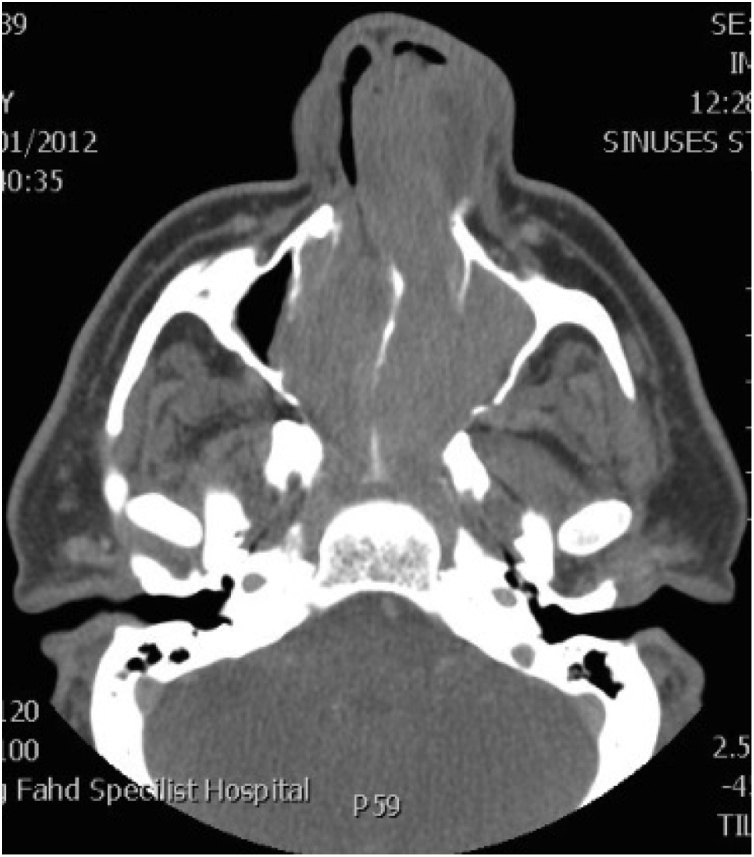
CT scan of the para-nasal sinuses showed bilateral homogenous opacity filling both nasal cavities and the left maxillary and frontal sinuses.

**Fig. 4:**
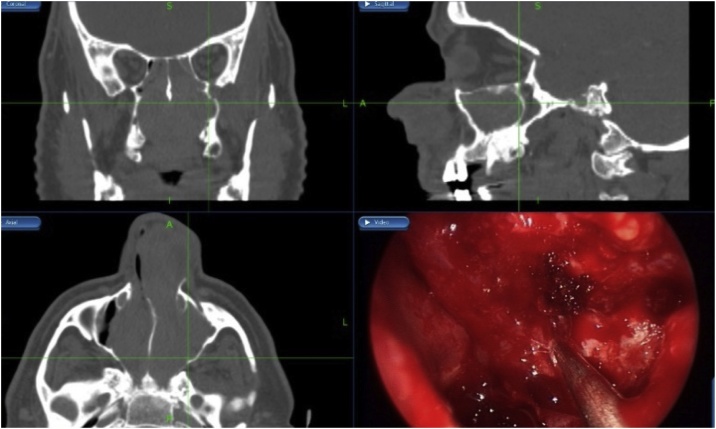
Patient underwent endoscopic navigation assisted bilateral medial maxillectomy.

**Fig. 5:**
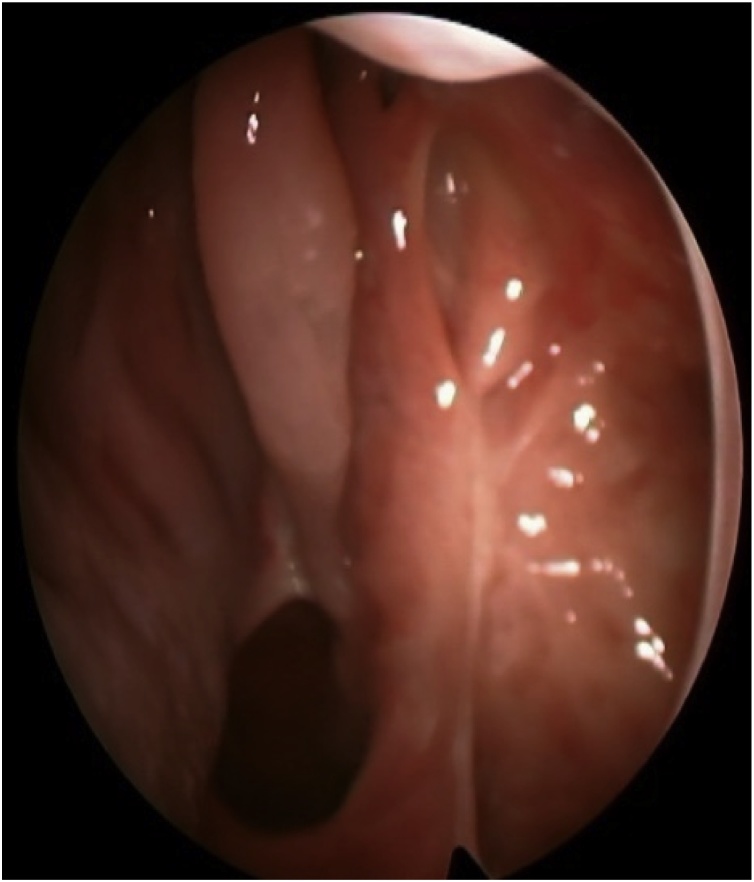
Post operation endoscopic view with no signs of recurrence.

A 58 years old healthy male. presented with history of left nasal obstruction started five years back then progressed to bilateral nasal obstruction associated with recurrent attack of left sided epistaxis. His physical examination findings; external nasal deformity, bulging left lateral nasal wall, fleshy mass seen through left nostril (Fig. 1), mass within floor of right nasal cavity. throat and ear examination were otherwise unremarkable. CT scan of the para-nasal sinuses showed bilateral homogenous opacity filling both nasal cavities and the left maxillary and frontal sinuses (Fig. 2, Fig. 3). Biopsy of both nasal masses revealed inverted papilloma. Patient underwent endoscopic, navigation assisted bilateral medial maxillectomy (Fig. 4). Histopathologic examination revealed sino-nasal papilloma with no evidence of dysplasia or surface erosion. The Patient remained symptoms free after the surgery. He has been following in the clinic for five years with no signs of recurrence (Fig. 5).

### 2.2 Case 2

65 years old male, a known case of DM, HTN, dyslipidemia. With past surgical history of multiple endoscopic sinus surgeries for recurrent nasal polyps. He presented to ENT clinic with history of bilateral progressive nasal obstruction for the past few months, associated with nasal discharges, and anosmia. He was found to have bilateral grade 4 nasal polyps. Throat, ear, head and neck examination were unremarkable.

**Fig. 6:**
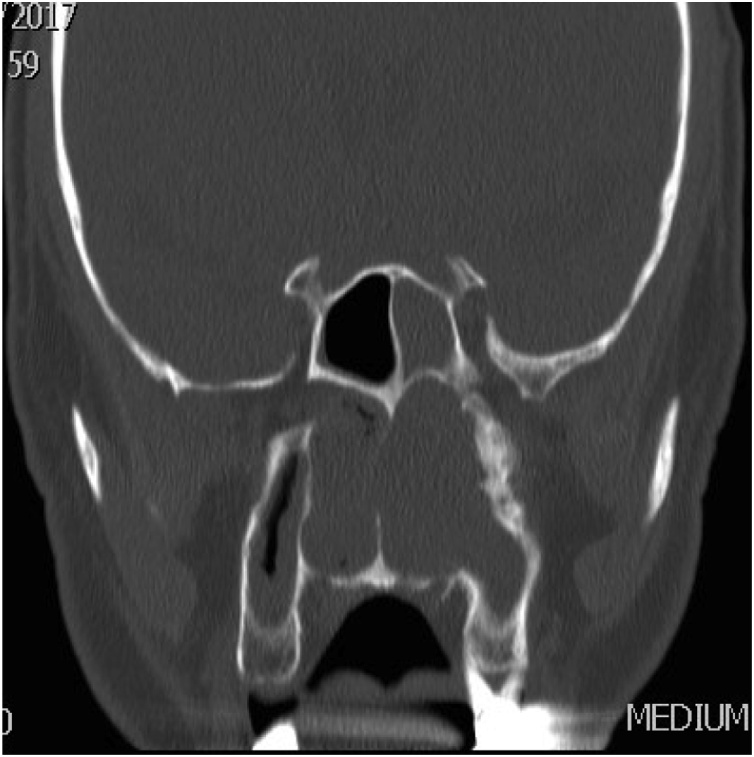
CT scan of the paranasal sinuses showed homogenous opacities filling both nasal cavities and the left maxillary, frontal and sphenoid sinuses.

**Fig. 7:**
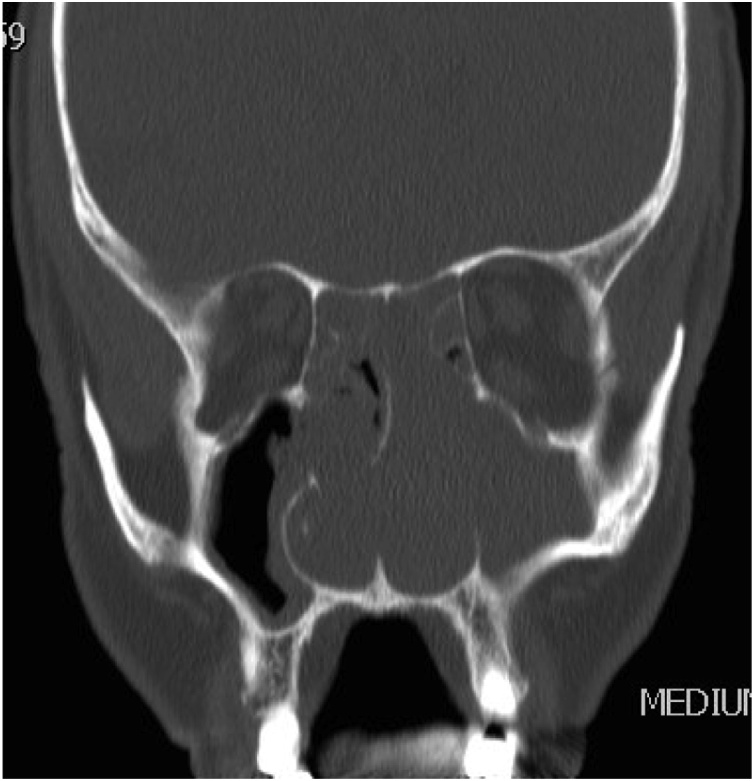
CT scan of the paranasal sinuses showed homogenous opacities filling both nasal cavities and the left maxillary, frontal and sphenoid sinuses.

**Fig. 8:**
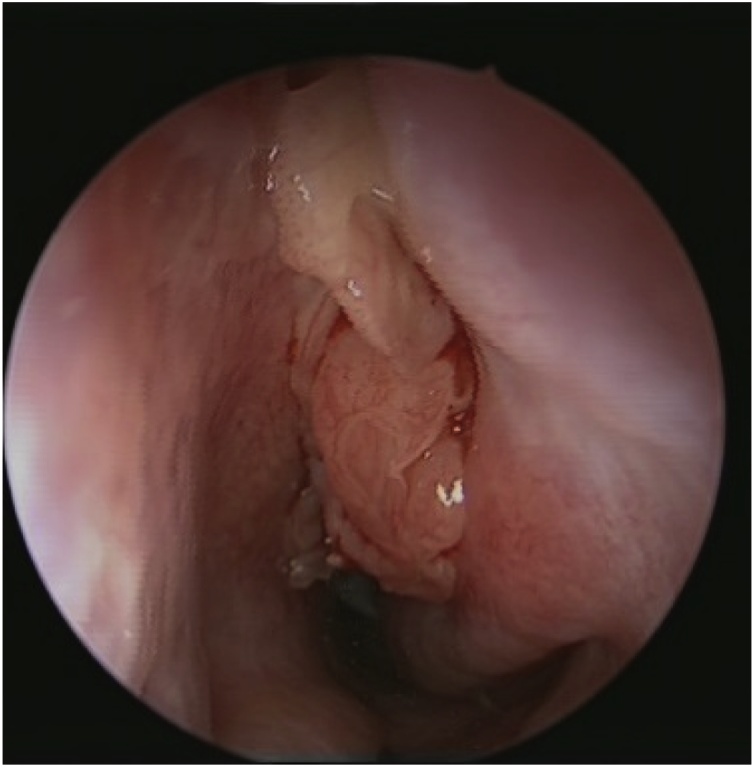
Biopsy of the polyps revealed bilateral inverted papilloma.

**Fig. 9:**
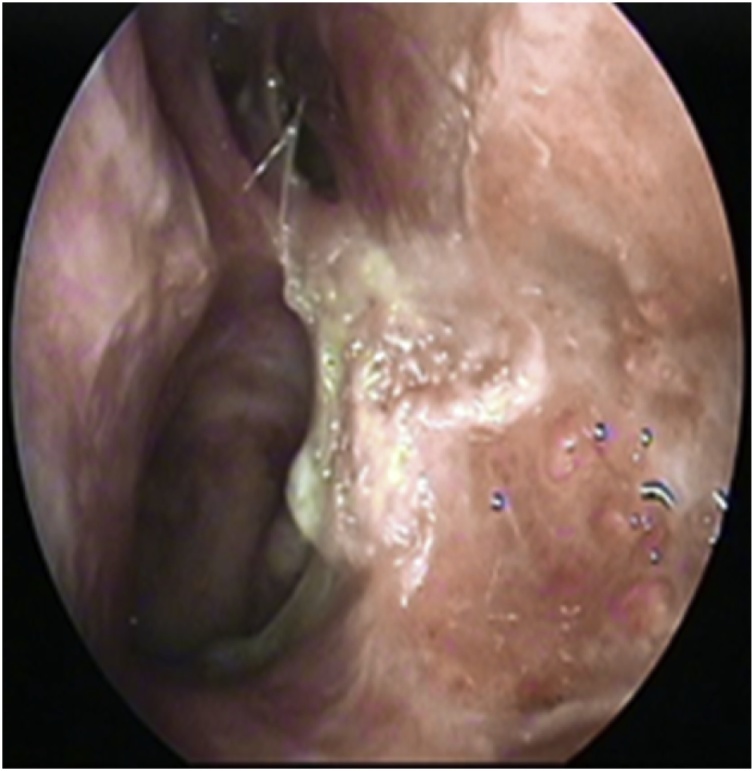
Endoscopic view Post bilateral endoscopic navigation assisted medial maxillectomies and tumor removal.

CT scan of the paranasal sinuses showed homogenous opacities filling both nasal cavities and the left maxillary, frontal and sphenoid sinuses (Fig. 6, Fig. 7). Biopsy of the polyps (Fig. 8) revealed bilateral inverted papilloma. Patient underwent bilateral endoscopic navigation assisted medial maxillectomies and tumor removal (Fig. 9).

The patient remained symptoms free, with no signs of recurrence for 3 years of follow up.

## 3 Discussion

Inverted papilloma (IP) is benign sinonasal tumor, the other variants of papilloma include the oncocytic and exophytic variants, it is the second most common benign sino-nasal tract tumor after osteoma [11]. usually take origin from the lateral nasal wall in the fontanelle area. The term inverted papilloma reﬂects its characteristic histological appearance of a covering epithelium that invaginates or inverts into the underlying stroma [9]. It is more common in men and usually presents in the ﬁfth and sixth decades of life [10]. The management of sinonasal IP is often complicated due to its local aggressiveness, high recurrence rate, and association with malignancy [11]. The clinical picture of IP is nasal obstruction, external nasal deformity, nasal discharges, PND, anosmia, facial heaviness, frontal headache and may be recurrent attack of epistaxis. CT scan is the initial modality of choice to evaluate the extent of disease [12]. Imaging also plays an important role in the preoperative assessment of inverting papilloma for assessment of the extent, configuration of the lesion and its relationship with surrounding structures, contrast enhanced MRI should also be considered as it has advantage over CT of better differentiating the tumor from inflammatory mucosal changes [12]. Krouse et al., has proposed a staging system for inverted papilloma which could help in the management plan of IP [22].

**Table:** 

T1	Tumor totally confined to the nasal cavity, without extension into the sinuses. The tumor can be localized to one wall or region of the nasal cavity, or can be bulky and extensive within the nasal cavity, but must not extend into the sinuses or into any extranasal compartment. There must be no concurrent malignancy
T2	Tumor involving the ostiomeatal complex, and ethmoid sinuses, and/or the medial portion of the maxillary sinus, with or without involvement of the nasal cavity. There must be no concurrent malignancy
T3	Tumor involving the lateral, inferior, superior, anterior, or posterior walls of the maxillary sinus, the sphenoid sinus, and/or the frontal sinus, with or without involvement of the medial portion of the maxillary sinus, the ethmoid sinuses, or the nasal cavity. There must be no concurrent malignancy
T4	All tumors with any extranasal/extrasinus extension to involve adjacent, contiguous structures such as the orbit, the intracranial compartment, or the pterygomaxillary space. All tumors associated with malignancy

Traditional simple intranasal removal was frequently followed by recurrence with a rate reported to be as high as 78 %. [23] this high rate likely indicates residual tumor reflecting inadequacy of the procedure, rather than actual recurrence as the rate of recurrence with the more advanced procedures is around 10 % [11]. Nonetheless, it also highlights the importance of long-term follow-up with regular endoscopic examination and or imaging.

Inverted Papilloma with bilateral involvement is rare, with a reported rate of up to 5 % [24]. The treatment of inverting papilloma is essentially surgical. Medial maxillectomy through lateral Rhinotomy was previously established as the gold standard for treatment of inverted papilloma, this technique was associated with potential aesthetic complications, endoscopic techniques have revolutionized the treatment with its clear advantage over open technique in terms of less hospital stay, improved post op pain and cosmetically with the absence of facial incision and minimal facial swelling [12]. However, an exclusively endoscopic approach may be contraindicated in the event of 1) massive involvement of the mucosa of the frontal sinus and/ or of a supraorbital cell; 2) transorbital extension, 3) concomitant presence of a malignancy that involves critical areas; and 4) presence of significant scarring and anatomic distortion from previous surgery [11].

Malignant transformation and concurrent malignant disease are well recognized in this benign sinonasal tumor with rates in the literature ranging from 6 to 14.5 % [18]. The risk factors for malignant transformation are yet to be understood. Eggers et al. [19] studied 93 cases of inverted papilloma with regards to both patient and tumor characteristics.

They concluded that certain histological features, namely increased mitotic count and dyscariosis, along with male gender and older age group put patients at a higher risk of malignant change.

Endoscopic surgery alone is not suitable for all cases. In one series of 104 cases when used in isolation the rate of recurrence was 22.4 % which dropped to 16.2 % when combined with open procedures for more extensive disease [17]. It has been proposed that endoscopic surgery alone is suitable for those lesions confined to the lateral nasal wall with or without extension into the ethmoid, maxillary and sphenoid sinuses [20] while tumors extending into the naso-frontal duct, orbit and frontal sinus may be better off with a combined procedure [20].

W. S. Kim et al. in a retrospective study on the treatment outcome of IP according to surgical approach suggested using an endoscopic approach in patients with Krouse stage 1 or 2, and mid-facial degloving approach for stage T3 as it has a lower recurrence rate compared with the endoscopic approach [25].

S.H. Ahn et al. in a retrospective study comparing the treatment outcome of IP using endoscopic approach with and without navigation assistance, and suggested the navigation assistance to be considered as it has helped reducing recurrence rate and surgical complications [26].

In our cases, as the lesions were not extensive or involving the naso-frontal duct, orbit and frontal sinus, endoscopic surgery alone was enough. Outcome studies have suggested that endoscopic and combined endoscopic/external approaches are at least equal in their effectiveness as more traditional techniques but are associated with a reduced hospital stay and decreased morbidity [21]. Fortunately, both cases did not show signs of recurrence on follow up

Q.Lisan et al. in a systemic review and meta-analysis investigating the potential association between the Krouse classification and the recurrence rates of sinonasal inverted papilloma, revealed a 51 % increased risk of recurrence for SIP classified as Krouse stage T3 disease when compared with stage T2 [27].

Risk factors for recurrence are still unclear, male sex which has an influence on incidence with a 3:1 male predominance, has been found to affect the rate of recurrence in several studies [28,1], history of previous sinus surgery, Tomazic et al.3 found a 50 % recurrence rate for revision surgery, compared with 12 % for primary resection, the endoscopic surgical approach is a safe and reliable option for IP when performed by an experienced surgeon, with several studies [29] showing that it has a superior recurrence compared with external approach, though controversy remains in this regard due to potential selection bias, M. Akkari et al. in a retrospective study of atypical presentations of IP including bilateral involvement and nasal polyp association, found that these atypical presentations had a higher recurrence rate but not reaching statistical significance, and suggested that the main determining factor in recurrence is the complete resection with attention to the site of attachment [1].

Both our presented cases, noticeably the second one was associated with nasal polyps and previous history of multiple sinonasal surgeries, all of which are factors associated with increased recurrence rate, both patients were managed successfully with complete endoscopic endonasal excision, with no sign of recurrence of follow up.

## 4 Conclusion

Bilateral involvement is an atypical, rare variation of inverted papilloma. Because of the high recurrence rate and potential of malignant transformation, IP is considered an aggressive invasive lesion. The definitive treatment is complete surgical excision. Endonasal Endoscopic approach is an effective, reliable and safe method of treatment, Careful preoperative planning coupled with meticulous surgical technique are absolute requisites for successful management of Inverted papilloma. Regular clinical follow up is essential after surgery to identify any sign of recurrence.

## Sources of funding

There is no financial support of sponsorship involved in this study.

## Ethical approval

Case reports are exempt from ethical approval at our institution.

## Consent

Written informed consent was obtained from the patient for publication of this case report and accompanying images. A copy of the written consent is available for review by the Editor-in-Chief of this journal on request

## Author contribution

Haifa Al Enzi: data collection, writing the original manuscript draft.

Mohammad AL Eid: review and editing of the manuscript, literature review.

Ali Al Momen: study concept, data analysis, final approval of manuscript.

## Registration of research studies

Not applicable

## Guarantor

Dr. Ali Al Momen

## Provenance and peer review

Not commissioned, externally peer-reviewed

## Data availability

The data used to support the findings of this study are included within the article. Also, they are available from the corresponding author upon request.

## Declaration of Competing Interest

The authors declare that there is no conflict of interest regarding the publication of this paper.
